# The Role of Oxidative Stress and Inflammation in Obesity and Its Impact on Cognitive Impairments—A Narrative Review

**DOI:** 10.3390/antiox12051071

**Published:** 2023-05-10

**Authors:** Ruth Naomi, Soo Huat Teoh, Hashim Embong, Santhra Segaran Balan, Fezah Othman, Hasnah Bahari, Muhammad Dain Yazid

**Affiliations:** 1 Department of Human Anatomy, Faculty of Medicine and Health Sciences, Universiti Putra Malaysia, Serdang 43400, Malaysia; gs60018@student.upm.edu.my; 2 Advanced Medical and Dental Institute, Universiti Sains Malaysia, Kepala Batas 13200, Malaysia; 3 Department of Emergency Medicine, Faculty of Medicine, Universiti Kebangsaan Malaysia, Kuala Lumpur 56000, Malaysia; 4 Department of Diagnostic and Allied Health Sciences, Faculty of Health and Life Sciences, Management and Science University, Shah Alam 40100, Malaysia; 5 Department of Biomedical Sciences, Faculty of Medicine and Health Sciences, Universiti Putra Malaysia, Serdang 43400, Malaysia; 6 Centre for Tissue Engineering and Regenerative Medicine (CTERM), Universiti Kebangsaan Malaysia, Kuala Lumpur 56000, Malaysia

**Keywords:** oxidative stress, obesity, adipose tissue, endogenous antioxidant, cognitive impairments

## Abstract

Obesity is a chronic low-grade inflammatory condition that induces the generation of oxidative stress and inflammation. This oxidative stress and inflammation stimulate brain atrophy and some morphological changes in the brain that eventually result in cognitive impairments. However, there is no exact study that has summarized the role of oxidative stress and inflammation in obesity and its impact on cognitive impairments. Thus, the objective of this review is to recapitulate the current role of oxidative stress and inflammation in cognitive decline based on in vivo evidence. A comprehensive search was performed in Nature, Medline and Ovid, ScienceDirect, and PubMed, and the search was limited to the past 10 years of publication. From the search, we identified 27 articles to be further reviewed. The outcome of this study indicates that a greater amount of fat stored in individual adipocytes in obesity induces the formation of reactive oxygen species and inflammation. This will lead to the generation of oxidative stress, which may cause morphological changes in the brain, suppress the endogenous antioxidant system, and promote neuroinflammation and, eventually, neuronal apoptosis. This will impair the normal function of the brain and specific regions that are involved in learning, as well as memory. This shows that obesity has a strong positive correlation with cognitive impairments. Hence, this review summarizes the mechanism of oxidative stress and inflammation that induce memory loss based on animal model evidence. In conclusion, this review may serve as an insight into therapeutic development focusing on oxidative stress and inflammatory pathways to manage an obesity-induced cognitive decline in the future.

## 1. Introduction

Obesity is a low-grade inflammation condition [1] known as metaflammation [2] with an excessive accumulation of fats and a body mass index (BMI) above 30 kg/m^2^. Obesity is classified as a multifactorial disease that usually arises when there is an excess intake of dietary needs with a low level of energy expenditure. In such a condition, the excess energy will be transformed into triglycerides, which will be then stored in adipose tissue. Over time, adipose tissue will increase in size (hypertrophy), causing the fat cells to increase in size, eventually evincing an increase in body weight [3]. One of the common factors leading to obesity could be the excessive intake of a high-fat diet (HFD). An HFD intake will stimulate the voluntary consumption of fat, leading to passive overeating. As so, in obese subjects, the regulation of fatty acid oxidation and lipid peroxidation will be dysregulated [4]. Hence, the manifestation of elevated oxidative stress and inflammation with a repressed antioxidant defense system is common [5]. Although the complications of obesity might differ from person to person, the consequences are similar for all subjects. This is true for the presence of oxidative stress and inflammation in all obese patients. One of the common overlooked complications of obesity is cognitive impairments. Obesity-related oxidative stress can cause some pathological changes to arise, such as brain atrophy, as well as inflammation that includes brain atrophy, suppressed hippocampal neurogenesis, reduced microvascular density, blood–brain barrier (BBB) dysfunction, and reactive astrogliosis, which may result in cognitive decline [6].

The recent global epidemiological data on obesity prevalence show that approximately 2.1 billion subjects are categorized either as overweight or obese. It is estimated that the United States government spent at least USD 190 billion in 2005 to treat obesity and its related complications [7,8]. The prevalence rate of obesity differs based on gender, geographical locality, and socioeconomic status [3]. As such, obesity is more common in older women compared to men [9], with a higher prevalence rate in the Asia Pacific region globally. Malaysia has recorded the highest prevalence rate for obesity in the ASEAN countries [10]. A report by the World Health Organization in the year 2021 reveals that at least 2.8 billion people worldwide die each year as a consequence of obesity [11]. Studies claim that obese patients are more prone to develop cognitive decline by 28% compared to lean subjects [12]. Recent discoveries have shown that a reduction in neural integrity in obese patients is one of the primary factors for cognitive impairments [13]. To add on, the endogenous antioxidant system such as glutathione (GSH), superoxide dismutase (SOD), and catalase (CAT) in obese patients is greatly suppressed, making obese patients more vulnerable to disease [14]. To date, there is no specific study that shows the interplay between oxidative stress and inflammation in obesity and how these could lead to cognitive decline. Thus, this review recapitulates the role of oxidative stress and inflammation in obesity as a cause of cognitive impairments. This study only focuses on animal models to rule out the direct link between obesity-associated oxidative stress in cognitive decline, as summarised in Table 1. The main reason why human studies were not taken into consideration is that human studies focus on obesity as a risk for neuropsychiatric disorders, such as dementia/autism/Alzheimer’s. In such circumstances, the complications of obesity such as diabetes, heart attack, or stroke can also lead to cognitive decline. Hence, the direct link between obesity-associated oxidative stress in cognitive decline in terms of mechanisms or signalling pathways involved could not be ruled out. Thus, to give a better understanding to the readers, the authors choose to focus only on the animal study, which gives a clear and concise explanation of the scope of this review.

## 2. Metabolic Changes in Obesity

Obesity is a chronic low-grade inflammatory condition that induces metabolic changes due to the excessive amount of adiposity. In obesity, excessive stress on the endoplasmic reticulum causes a multimer form of adiponectin to be formed, resulting in stimulation response from unfolded protein [15]. Adiponectin is a cytokine (a protein hormone) released by adipocytes. Adiponectin plays a vital role in reducing inflammation and preventing the deposition of fats in the arteries [16]. Thus, the inhibition of adiponectin will cause the circulatory level of adiponectin to decrease [15]. When the amount of free fatty acids is more than what the adipocytes can retain, the production of adiponectin is also inhibited. Since adiponectin controls the inflammatory response by causing the formation of nitric oxide through vascular endothelium, a lower amount of the hormone will also limit its anti-inflammatory effects [17]. One of the primary functions of adiponectin is to regulate lipid metabolism by modulating the transfer of free fatty acids and β oxidation into the muscle, thereby preventing hepatic lipogenesis. As a result, those fatty acids will be stored in adipose tissue, causing the circulating lipids to decrease [18]. However, in obesity, adiponectin raises the influx and combustion of free fatty acids in the muscles, causing the level of triglycerides in the muscle to reduce and increase in the plasma [19]. Thusly, the free fatty acids will enter the hepatic portal, activating the signaling pathway of gluconeogenesis and lipid synthesis. This process will inhibit the signal transduction for insulin and manifest as insulin resistance in the peripheral [20].

Another common metabolic change observed in obesity is increased leptin concentration in the plasma. Generally, white adipose tissue produces leptin and it exhibits the effects either via the nervous system or directly via the activity of autocrine signaling [21]. Leptin is a hormone that controls satiety. In obesity, an increased concentration of leptin is due to the excessive level of adipocytes that impairs the leptin signaling receptor, developing a condition known as leptin resistance. As a consequence, the ability of leptin to feel a sense of satiety is lost, leading to decreased energy expenditure and overconsumption of nutrients [22]. Leptin is also involved as a regulator in neuroendocrine signaling and the immune system due to its ability to increase the production of pro-inflammatory factors in the acute phase of inflammation [23]. However, as a feedback loop, the leptin concentration in adipose tissue will be further increased in the presence of inflammatory factors, such as TNF-α and IL-1. Thus, the level of inflammation will also increase in obesity due to hyperplasia and hypertrophy of adipose tissue [24].

Another common pathological change observed in obesity is the increased production of endotrophin [25]. Endotrophin is an extracellular matrix derived from the collagen VI subunit of α3 [26]. Endotrophin stimulates the formation of transforming growth factor (TGF)-β [27], promoting the activity of adipogenesis [28]. Excessive concentration of endotrophin might stimulate the formation of fibrotic deposition of collagen, macrophage infiltration, and inflammation within the dysfunctional adipocytes, leading to insulin resistance [25].

**Table 1:** The in vivo models of obesity-induced cognitive decline.

Author	Model, Strain, and Gender	Mode of Obesity Induction and Duration	Test to Access Cognition	Findings
Karimi et al., 2013 [29]	180–200 g of adult male Wistar rats	High-fat diet for 26 weeks	Not specified	- The body weight had increased drastically. - The excitatory post-synaptic potentials (EPSP) slope had decreased. - The population spike (PS) amplitude had decreased.
André et al., 2014 [30]	3-week-old male C57BL/6J mice	Western diet for 20 weeks	- Y maze - Elevated plus maze (EPM) - Tail-suspension test (TS) - Forced swim tests	- The body weight had increased drastically. - The level of leptin in adipose tissue and the serum had increased. - The random exploration in all arms of the Y maze was noticed. - The time spent exploring the open arms of EPM had decreased. - The depressive-like behavior in TS had increased. - The time in finding an escape platform in the forced swim test had increased. - The level of leptin, insulin, resistin, and lipopolysaccharide in plasma had increased. - The level of TNF-α, IL-6, and adipokines in plasma had increased. - The level of the kynurenine/tryptophan ratio in the lung had increased. - The level of TNF-α and IFN-γ in the hippocampus had increased. - The level of IL-1β, IL-6, and the suppression of cytokine signaling 3 (SOCS3) in the hypothalamus had increased.
Jayaraman et al., 2014 [31]	3-month-old male C57BL6 mice	High-fat diet for 16 weeks	Not specified	- The body weight had increased drastically. - The level of TNF-α and IL-1β in the cerebrocortical of the brain had increased. - The level of neurites and their length in the glial cells had decreased. - The level of macrophage infiltration in the sciatic nerve had increased.
Erion et al., 2014 [32]	5-week-old male C57BL/6/J	Homozygous leptin receptor mutant mice	- Treadmill running - Y maze - EPSP	- The body weight had increased drastically. - The physical activity regarding treadmill training had decreased. - The random exploration in all arms of the Y maze was noticed. - The EPSP slope had decreased. - The impairment of dentate gyrus long-term potentiation (LTP) was noticed. - The level of IL-1β, microglial, and macrophage infiltration in the hippocampus and adipose tissue had increased. - The level of the serum and leptin had increased consistently.
Tucsek et al., 2014 [33]	7–24-month-old male C57BL/6 mice	HFD for 5 months	- EPM - Grip strength test	- The body weight had increased. - The transfer latency period had increased. - The level of CA1 in the hippocampus and retrosplenial cortex had decreased. - The level of pericytes coverage in the hippocampal microvessels had decreased. - The level of the angiogenic gene in the hippocampal region had increased. - The level of cerebral blood flow in the brain had decreased. - The level of NADPH oxidase and 3-nitrotyrosine in the cortex of the brain had decreased.
Liu et al., 2014 [34]	19–22 g male C57BL/6J mice	HFD for 20 weeks	- Morris water maze (MWM) - Step through the passive avoidance test	- The body weight had increased. - The mean latency escape period in MWM and stepThrough pass avoidance test had decreased. - The plasma insulin and fasting blood glucose level (FBG) had increased. - The level of glutathione (GSH) and superoxide dismutase (SOD) in the cortex and hippocampus had decreased. - The level of malondialdehyde (MDA), TNF-α, IL-6, and NF-κB in the cortex and hippocampus had increased. - The level of monocyte chemoattractant protein-1 (MCP-1) and resistin in plasma had increased. - The level of synapsin I, postsynaptic density protein 95, and the brain-derived neurotrophic factor (BDNF) in the cortex and hippocampus had decreased. - The neuronal atrophy in the CA1 region of the hippocampus had increased.
Bocarsly et al., 2015 [35]	Male Sprague–Dawley (SD) rats	HFD for 8 weeks	- Novel object recognition test (NORT) - place recognition test - Attentional set shifting (ASST)	- The body weight, abdominal, and gonadal fat had increased. - The level of triglycerides, insulin, and leptin in plasma had increased. - Lower discrimination ratios in object and place recognition were noticed. - The mean errors before reaching the criterion in ASST had increased. - The level of dendritic spine density in the CA1 region of the hippocampus had decreased. - The length of secondary and tertiary microglial processes in layer I in the prefrontal cortex had increased.
Madhavadas et al., 2015 [36]	18-month-old male SD rats	Monosodium glutamate was injected intraperitoneally daily for 14 days	- Barnes maze task	- The body weight had increased. - The nasoanal length had decreased. - The level of glucose, cholesterol, and leptin in the serum had increased. - The level of acetylcholinesterase activity in the hippocampus had increased. - The level of hippocampal weight had decreased. - The spatial disorientation score in the Barnes maze task had increased.
Wang et al., 2015 [37]	4-week-old male C57BL/6 mice were	HFD for 20 weeks	- NORT - MWM	- The level of recognition index in NORT had reduced. - The timing in the escape latency period in MWM had increased. - The level of glucose, insulin, free fatty acids, and cholesterol in plasma had increased. - The level of reactive oxygen species (ROS) in the hippocampal mitochondria had increased. - The level of adenosine triphosphate (ATP), mitochondrial membrane potential, and pAMPK172 in the hippocampal mitochondria had decreased.
Pratchayasakul et al., 2015 [38]	200–220 g weighing female Wistar rats	HFD for 12 weeks	- MWM - Open-field test	- The body weight and visceral fat had increased. - The timing in the escape latency period in MWM had increased. - The level of glucose, cholesterol, triglyceride, HDL, LDL, insulin, and HOMA in plasma had increased. - The level of tyrosine phosphorylation of IR (pIR) and Akt/PKB phosphorylation at the serine 473 site in brain slices had decreased. - The level of ROS, MDA, unfolding, and swelling in brain mitochondria had increased. - The level of LTP and density of dendritic spines in the CA1 region of the hippocampus had decreased.
Hargrave et al., 2015 [39]	275–300 g weighing male SD rats	High-fat diet and high dextrose western diet for 3 months	- Y maze	- The weight gain and calorie intake had increased. - The random exploration in all arms of the Y maze was noticed. - The engagement in vicarious trial and error in the Y maze had decreased. - The level of insulin, glucose, and β-hydroxybutyrate in plasma had increased. - The level of glucose transporter 1 (GLUT1) and monocarboxylate in the hippocampus had decreased.
Stranahan et al., 2016 [40]	6-week-old male leptin receptor mutant mice	Not specified	- Y maze - NORT	- The body weight, inguinal, epididymal, and interscapular fat had increased. - The random exploration in all arms of the Y maze was noticed. - The level of recognition index in NORT had reduced. - The BBB integrity in the brain had reduced. - The level of claudin 5 and occluding in the hippocampus had decreased. - The impairment of dentate gyrus LTP was noticed. - The macrophage infiltration in the forebrain and CD11b in microglial cells had increased. - The level of IL-1, IL-6, MCP1, and TNF-α in the plasma had increased.
Fu et al., 2016 [41]	2-month-old adult SD rats	HFD for 6 months	- Y maze - NORT	- The random exploration in all arms of the Y maze was noticed. - The level of recognition index in NORT had reduced. - The level of triglycerides and total cholesterol in plasma had increased. - The level of insulin in plasma and the hippocampus had increased. - The expression of Akt and ERK1/2 in the hippocampus had increased.
Sa-nguanmoo et al., 2016 [42]	200–220 g weighing male Wistar rats	HFD for 12 weeks	- MWM - Open-field test	- The body weight had increased. - The timing in the escape latency period in MWM had increased. - The level of total cholesterol, low-density lipoprotein (LDL), insulin, and glucose in plasma had increased. - The level of TNF-α and MDA in the serum had increased. - The level of adiponectin in plasma had decreased. - The impairment of dentate gyrus LTP was noticed. - The level of dendritic spine density in the CA1 region of the hippocampus had decreased. - The level of fibroblast growth factor 21 in the plasma had increased. - The level of phosphorylated ERK1/2 and peroxisome proliferator-activated receptor γ coactivator (PGC1-α) in the brain had decreased. - The level of ROS formation and swelling in the brain mitochondria had increased. - The lipid peroxidation and Bax in the brain had increased. - The level of Bcl-2 in the brain had decreased.
Jais et al., 2016 [43]	APP.PS1-transgenic mice	HFD for 16 months	- MWM	- The timing in the escape latency period in MWM had increased. - The expression of *Slc2a1* and GLUT1 in the brain had decreased. - The level of VEGF and non-esterified fatty acids in the serum had increased. - The level of glucose uptake in the cortex, hypothalamus, and nucleus accumbens of the brain had decreased. - The integrity of the BBB in the brain had decreased. - The level of activated astrocytes in the cortex and hippocampus of the brain had increased.
Mi et al., 2017 [44]	3-month-old C57BL/6J mice	HFD for 16 weeks	- MWM	- The body mass and fat content had increased. - The timing in the escape latency period in MWM had increased. - The leptin, insulin, resistin, and glucose level in the plasma had increased. - The pyknotic nuclei in the hippocampus had increased. - The level of mRNA expression of *Nsf* and *Vamp1* in the hippocampus had increased. - The phosphorylation of Tyr612 had increased and the phosphorylation of Ser307 in the brain had decreased. - The expression of GSK3β and GLUT1, 3, and 4 in the brain had decreased. - The expression of phosphorylated p38, NF-κB, and TNF-α in the brain had increased. - The expression of BDNF, NT-3, NT-4, and NGF in the brain had decreased.
Arnoldussen et al., 2017 [45]	Male LDLr Leiden mice	HFD for 15 weeks	- MWM	- The body weight, liver weight, omental, epididymal, and inguinal fat deposition had increased. - The level of triglycerides and cholesterol in the plasma had increased. - The fat tissue size (hypertrophy) fibrosis and macrovesicular steatosis in the liver had increased. - The timing in the escape latency period in MWM had increased. - The level of cerebral blood flow in the thalamus and hippocampus had decreased. - Increased activation of microglial cells in the thalamus and hippocampus had increased.
Nameni et al., 2017 [46]	250–300 g weighing male Wistar rats	HFD for 16 weeks	- MWM	- The body mass had increased. - The timing in the escape latency period in MWM had increased. - The level of glucose and insulin in plasma and cerebrospinal fluid had increased.
Tarantini et al., 2017 [47]	12-week-old Male wild type mice (Nrf2+/+) and Nrf2 KO mice	HFD for 5 months	- NORT - EPSP	- The level of glucose, total cholesterol, triglycerides, IL-6, MCP-1, TNF-α, IL-1β, and 1L-12 in the plasma had increased. - The level of recognition index in NORT was reduced. - The level of p16^INK4A^ and activated microglial in the brain had increased. - The BBB integrity had decreased. - The EPSP slope had decreased. - The impairment of dentate gyrus LTP was noticed.
Miranda et al., 2017 [48]	9-week-old male C57BL/6J mice	HFD for 13 weeks	- MWM	- The body and liver weight had increased. - The timing in the escape latency period in MWM had increased. - The level of leptin, insulin, glucose, alanine transaminase (ALT), and aspartate aminotransferase (AST) in the plasma had increased. - The expression of AMPK in the liver had decreased.
Manchanda et al., 2017 [49]	3–4-month-old female Wistar albino rats	HFD for 12 weeks	- Rotarod test - Narrow beam walk test - NORT	- The body weight had increased. - The level of recognition index in NORT was reduced. - The range of rearing behavior had decreased. - The level of polysialylated neural cell adhesion molecules in the brain had increased. - The level of calcium and calmodulin-dependent protein kinase in the brain had decreased. - The phosphorylation of Akt-1, c-JUN, c-FOS, BDNF, and tropomyosin receptor kinase B (TRKB) in the hippocampus had decreased.
Cope et al., 2018 [50]	8-week-old adult male C56BL/6J mice	HFD for 10 weeks	- NORT - Barnes maze test	- The body weight had increased. - The level of recognition index in NORT was reduced. - The longer path in the Barnes maze test was noticed. - The level of dendritic spine density in the CA1 region of the hippocampus had increased. - The microglial activation in the brain had increased.
Duffy et al., 2018 [51]	7–8-month-old male wild type and orexin ataxin-3 mice	HFD for 28 days	- Two-way active avoidance task - Open-field test	- The body weight had increased. - The avoidance and increased latency period were reduced. - The expression of orexin 1 receptor, Iba1, TNF-α, CX3CR1, and Irg1 in the hippocampus had increased.
Chunchai et al., 2018 [52]	180–200 g weighing male Wistar rat	HFD for 12 weeks	- MWM	- The body weight and plasma glucose had increased. - The level of IL-6 and IL-1 in plasma had increased. - The EPSP slope had decreased. - The impairment of dentate gyrus LTP was noticed. - The mitochondrial swelling, ROS formation, and depolarization had increased. - The microglial activation in the CA1 region of the hippocampus had increased. - The timing in the escape latency period in MWM had increased. - Less time spent at the escape platform was noticed.
Jeong et al., 2019 [53]	5-week-old specific pathogen-free male C57BL/6 J mice	HFD for 9 weeks	- Y maze - Elevated plus maze test - NORT	- The body mass had increased. - The CD11b^+^/CD11c^+^ infiltration in colonic epithelium had increased. - The level of recognition index in NORT was reduced. - Random exploration in all arms of the Y maze was noticed. - The anxiety-like behavior had increased. - The level of BDNF and CREB in the hippocampus had decreased. - The expression of NF-κB and Iba1 in the hippocampus had increased.
Shi et al., 2020 [54]	11-week-old C57Bl/6 J male mice	HFD for 15 weeks	- NORT	- The body weight had increased. - The level of recognition index in NORT was reduced. - The colonic epithelial cells and mucosal content had decreased. - The level of occludin and zonula occludens-1 in the colon had decreased. - The colon length had decreased. - The level of TNF-α, IL-6, and IL-1β in the serum and hippocampus had increased. - The BBB integrity and increased activation of microglial cells in the brain had decreased. - The expression of Iba1 and GFAP in the hippocampus had increased. - The level of PTP1B and IRS-1 in the hippocampus had increased. - The level of synapsin I, synaptophysin, and postsynaptic density 95 in the hippocampus had decreased.
Shi et al., 2020 [55]	11-week-old C57Bl/6 J male mice	HFD for 15 weeks	- NORT - Place recognition test	- The object and place discrimination index had decreased. - The microglial activation, Iba-1, and PSD95 in the hippocampus had increased. - The level of TNF-α, IL-1β, and IL-6 in the hippocampus had increased. - The level of PTP1B and IRS-1 in the hippocampus had increased. - The level of colonic mucosal content and mucin-2 glycoprotein in the colon had decreased.

## 3. Obesity and Brain Morphological Changes

The changes in brain morphology have a positive influence on cognitive impairment, and an increase in body weight is said to influence the changes in brain volume. Studies show that high BMI can cause brain atrophy of the temporal lobe, the second largest lobe that operates auditory information [6]. The density of the grey matter at the middle and post-central gyrus, putamen, and frontal operculum decreases with an increase in body mass [56]. The volume of the hippocampus tends to reduce with increased body weight, thereby causing memory impairment over time. Since the hippocampus has a vital function in learning and memory, the volume reduction will disrupt the synaptic transmission and impair LTP in the dentate gyrus [29] and CA1 region [57], resulting in poor memory. Neuronal apoptosis and poor neurogenesis mechanisms in the morphological changes are commonly observed in the hippocampus of obese subjects [6].

Another study shows that some obesity-associated morphological changes are present in male subjects. For instance, the grey matter volume is drastically reduced in the posterior lobe of the cerebellum, the perisylvian regions of temporal lobes, and the bilateral orbito-frontal gyrus in obese male subjects, which was not observed in the obese female subjects [58]. The changes in brain volume are mainly due to the impaired insulin transport to the brain that often arises in the case of obesity. Impaired insulin receptors will result in impaired insulin sensitivity, thereby hindering the transport of insulin to the brain and diminished glucose usage for neuronal survival, eventually leading to abnormal axons and myelin structure in the frontal lobe, causing shrinkage of the brain [59]. To boost, the high level of adiposity may induce inflammation in the brain, overstimulating gliosis and eventually making the cortisol of the brain become thinner [60]. All of these changes will manifest as poor learning and memory in due time.

### 3.1. Oxidative Stress in Obesity

One of the pathological changes recorded in obesity is the excessive amount of ROS and oxidative stress. The main source for ROS is either through oxidase, such as nicotinamide adenine dinucleotide phosphate (NADPH), or via the mitochondrial respiratory chain. In obesity, mitochondria in adipocytes serve as one of the main sources of ROS formation. During the mitochondrial oxidative phosphorylation, O_2_ will be reduced to H_2_O, causing the electron to enter the flavin mononucleotide and ubiquinone cycle interact with the O_2_ molecules, generating superoxide anion (O_2_^−^) and hydrogen peroxide (H_2_O_2_) in the mitochondria [61]. One of the ligand-activated transcription factors involved in the formation of ROS in obesity includes the peroxisome proliferator-activated receptor (PPAR) and its downstream mediators.

#### 3.1.1. Peroxisome Proliferator-Activated Receptors

The PPAR is known as a ligand-responsive nuclear receptor, and its receptor has three families: PPAR-α, PPAR-β, and PPAR-γ. The PPAR receptor is activated by endogenous ligands, such as fatty acids and their metabolites. Thiazolidinediones are one of the agonists for the PPAR and it will stimulate lipid storage in adipocytes by reducing lipotoxicity in the liver and skeletal muscles. This PPAR agonist prevents the development of obesity and is related to insulin resistance in rodents as well. Serious inflammation in white adipose tissue (WAT) may lead to macrophage infiltration, which is linked to insulin resistance. Adipocytes are stimulated to release chemokines by proinflammatory cytokines, which can aid in the infiltration of macrophages. The PPAR plays a critical role in fatty acid catabolism in the liver by boosting the expression of numerous genes involved in mitochondrial fatty acid oxidation, peroxisomal fatty acid oxidation, and various other aspects of fatty acid metabolism in the cell. As a result, PPAR activation can stop and lessen the development of hepatic fat [26,52,62].

#### 3.1.2. Prostaglandin E2

Prostanoids are bioactive substances that are generated in response to numerous stimuli and are crucial for tissue homeostasis and inflammation. They are composed of prostaglandin and thromboxane. One of the main prostanoids produced by the cyclooxygenase (COX) and PGE synthase metabolism of arachidonic acid is prostaglandin E2 (PGE_2_). Its four G protein-coupled receptors are EP1 through EP4. PGE_2_ blocks the synthesis of inflammatory cytokines and chemokines in LPS-treated human and murine macrophages via EP4. The activation of EP4 inhibits chronic inflammation in living organisms by lowering the activation of macrophages throughout the disease. EP4 signaling stops the growth of adipocytes and protects mice from streptozotocin’s harmful effects that cause diabetes. PGE_2_ at least partially mediates the physiologic effects of adiponectin (an important anti-inflammatory and anti-atherosclerotic molecule that suppresses adipocyte differentiation and inhibits ischemia-reperfusion injury). PGE_2_ prevents apoptosis and regulates the profibrotic and proinflammatory activation of pancreatic stellate cells caused by EP4. In obese patients, macrophages not only attack adipose tissues but also the liver and skeletal muscles, and the polarity appears to have shifted toward M1, which has a strong link with insulin resistance and organ failure [63,64,65].

#### 3.1.3. Cyclooxygenase

The rate-limiting COX has two isoforms: COX-1, which is constitutive, and COX-2, which is mostly synthesized by prostaglandins (PGs). The main enzyme in eicosanoids’ metabolism, COX-2, converts eicosanoids into several PGs, including prostacyclin (PGI2), PGD2, PGE_2_, and PGF2. The COX-2 gene and immunoreactive proteins have been demonstrated to be highly expressed and elevated in adipose tissue in conditions of morbid obesity. However, it has been shown that environmental stress-induced expression and constitutive overexpression of COX-2 play a variety of roles under different clinical and physiological situations; for instance, the overexpression of the COX-2 gene in white WAT. It also plays an important role in obesity-related metabolic syndrome. COX-2-derived PGs also significantly contribute to the control of energy metabolism under various pathophysiological circumstances. Arachidonic acid is released from membrane phospholipids by phospholipase A2 in response to inflammatory stimuli, which is the first step in the creation of PGs. The cyclooxygenase enzymes COX-1 and COX-2 transform arachidonic acid into PGH2 [63,66,67].

#### 3.1.4. Mitogen-Activated Protein Kinase (MAPK)

Mitogen-activated protein kinase (MAPK) pathways are signaling pathways that transmit extracellular and intracellular signals that are involved in regulatory networks within the cell. MAPK kinase (MAPKK) and MAPK kinase are gradually activated by extracellular stimuli that start each cascade, activating the MAPKK. The three primary groups of MAPK are extracellular signal-regulated kinases (ERKs), Jun amino-terminal kinases (JNKs), and stress-induced protein kinases (p38/SAPKs). While the ERKs primarily react to stresses, such as ionizing and increased lipid peroxidation to stimulate cell growth and differentiation, the JNKs are essential in apoptosis, cytokine secretion, inflammation, and metabolism. Cellular stress, cytokines, and the p38 MAPKs all significantly activate them. The p38 MAPK pathway is associated with the control of the cell cycle, apoptosis, cellular proliferation, and inflammation in adipogenesis [68,69].

#### 3.1.5. Phosphoinositide 3-Kinase (PIK3CA—AKT) Pathway

Through all the PI3K/AKT signaling pathways, lipolysis is inhibited, and lipid production is increased. SREBP, which controls lipids synthase and fat genes, which control lipolysis by modulating the production of adipose triglyceride lipase, are two of the primary substrates for AKT-mediated lipid accumulation. In the feeding state, SREBP-1c is controlled by four pathways: activated AKT stimulates the liver X receptor, a stipulation for SREBP-1c translation; activated AKT stimulates mTORC1 to activate SREBP-1c transcription; mTORC1 also prevents Lipin-1, reducing the half-life of nuclear SREBP-1c; and induced AKT stimulates S6K1, which promotes SREBP-1c maturation and activates it. When adrenergic signaling causes cAMP to increase causing hormone-sensitive lipase (HSL) and perilipin to be activated by PKA, acute lipolysis is induced during a fast. In the fed state, PI3K/AKT inhibits protein kinase A (PKA), which stops lipolysis. The AKT-independent and PI3K-dependent pathways both control the rate-limiting lipolytic enzyme, which is responsible for triacylglycerol hydrolase activity and transcription factor 4 (IRF4), which helps promote lipogenesis, in part, by triggering the expression of the lipases, as well as HSL and AKT-mediated lipid accumulation [54,55,61].

#### 3.1.6. Protein Kinase C

The lipid-activated serine/threonine PKC family of enzymes, which contains protein kinase C (PKC), has been linked to a variety of important physiological systems. The novel function of PKC in modulating mitochondrial function to control triglyceride homeostasis. PKC is a corepressor that regulates the homeostasis between power use and total energy. Genes that affect the oxidation of fatty acids and proteins involved in energy loss are expressed at higher levels. It has been shown that alterations in adipose PKC expression are essential for diet-induced obesity and accompanying metabolic abnormalities. It was shown that an HFD particularly impacts specific isoforms of white adipose tissue despite having no impact on some other tissues. Genetically modified mice lacking PKC are leaner with greater oxygen consumption, resistant to obesity and steatosis put on by HFD, and have enhanced insulin sensitivity. Whenever an HFD stimulates PKC in adipose tissue, it alters mitochondrial homeostasis by intersecting with p66Shc signaling, increases adipose dysfunction, and has adverse effects. PKC acts as a “diet-sensitive” metabolic sensor [63,67].

### 3.2. Inflammation in Obesity

The level of immune tolerance is greatly suppressed in obesity and this leads to the excessive secretion of inflammatory mediators such as IL-1β, IL-6, TNF-α, and IFN-γ and a drastic reduction in anti-inflammatory factors, such as IL-4 and IL-10 [62]. Pro-inflammatory factors, such as TNF-α, enhance hyperlipidemia by stimulating the lipid to synthesize in the liver. At the same time, TNF-α prevents the removal of triacylglycerol and insulin-activated de novo lipogenesis [70].

#### 3.2.1. Interleukin 2

Cytokines associated with fatty tissue have a role in the onset of persistent low-grade inflammation in obese individuals. Interleukin 2 (IL-2) is a pleiotropic cytokine that plays a function in immunological changes that take place during inflammation. IL-2 is produced by the activated T cells that enter adipose tissue in obesity. Using RNA from adipose tissue isolated from overweight and obese people shows that transcript/protein levels of IL-8, IL-12A, CCL5, CCL19, CCR2, and CCR5 are related to IL-2 mRNA/protein levels in the AT. As a result, it implies that obesity may be a prediction of AT malfunction, which is linked to monocyte and macrophage invasion and the production of IL-2 by active T cells. Since IL-2 expression is independently correlated with that of IL-8 and IL-12A, the latter is likely essential in fostering the production of the former cytokines. It was discovered that concurrent TNF and IL-8 expression in the AT was related to macrophage infiltration into the fat. Significant increases in the production of IL-2, IL-8, IL-12A, CCL5, CCL19, CCR2, CCR5, TLRs, and other variables in the AT suggest that these elements may interact to induce insulin resistance in this population. In obesity, the expression of the AT IL-2 gene and protein was elevated, and it was highly associated with indicators of inflammation, metabolism, and insulin resistance [68,70].

#### 3.2.2. IL-8

The main producers of IL-8, which is important in regulating the inflammatory response, are monocytes and macrophages. Inflammation may have an impact on the genesis of atherosclerosis, and IL-8 is probably also an atherogenic factor. Oxidized LDL particles can promote the production and release of IL-8 by macrophages from human atherosclerotic plaques, as well as produce high levels of IL-8 in human foam cells. Another way that IL-8 functions is by preventing the expression of tissue inhibitors of metalloproteinase. By increasing the release of metalloproteinases that degrade the matrix, this activity promotes the instability of atherosclerotic plaque. Adipocyte IL-8 mRNA expression, protein synthesis, and release, which are stimulated by IL-1 and IL-6, were also found to be inhibited by the insulin-sensitizing agents [68,69,70].

#### 3.2.3. IL-6

IL-6 is a cytokine that contributes to the control of inflammatory reactions and stimulates inflammation. It is an endocrine cytokine that mostly affects areas other than the one it began with because of the membrane-bound receptor, to which it binds IL-6R. Obese patients were shown to have higher serum levels of IL-6, as well as those with chronic inflammatory diseases and abnormal blood lipid values. Obese individuals may have higher levels of IL-6, which may increase their chance of developing type 2 diabetes, insulin resistance, and cardiovascular complications. The vascular difference of IL-6 across abdominal subcutaneous adipose tissue under basal conditions showed that IL-6 is released into the bloodstream in a concentration sufficient to affect the endocrine system. Adipose tissue was shown to produce a third of the total amount of circulating IL-6. If the number of adipocytes is adjusted, obese adipose tissue from obese people expresses IL-6 at a higher level than adipose tissue from lean people. The hypothalamus has a higher expression of IL-6 receptors, which suggests that IL-6 may be involved in controlling appetite and calorie intake [69,70].

#### 3.2.4. LDH

The LDH enzyme catalyzes the conversion of lactate to pyruvate, which is a crucial step in the cellular energy production process. Among organs having relatively high LDH levels are the muscles, the heart, the kidneys, and the liver. The five LDH isoenzymes are LDH-1, LDH-2, LDH-3, LDH-4, and LDH-5. In general, each isoenzyme is used most frequently by the cells in a certain tissue. The heart is the primary location of LDH-1. The body’s resistance to infection is mostly tied to LDH-2 (reticuloendothelial system). The liver and striated (skeletal) muscles have LDH-3, the kidneys, placenta, and pancreas have LDH-4, and the lungs and other tissues include LDH-5. Usually, the concentration of LDH-2 is higher than that of the other isoenzymes. Another enzyme of relevance is LDH, which plays a significant part in anaerobic metabolic processes. LDH may act as a biomarker for metabolic syndrome, specifically for obesity [63,70].

#### 3.2.5. Monocyte Chemotactic Protein 1

An expression of MCP-1 is present throughout all types of cells and is triggered by a variety of stimuli. MCP-1 overexpression is linked to insulin resistance, as well as macrophage activation and deposition in adipose tissues, in obese individuals. MCP-1 expression is highest in the stromal vascular component of white adipose tissue, which is attributed to the presence of macrophages. Some preadipocytes exhibit slightly elevated MCP-1 expression when TNF is activated. Obese adults and obese children usually have plasma levels of MCP-1 that are higher than the lean controls. It is related to the number and size of omental adipocytes in newborns, and visceral obesity has a positive effect in adults. Fructose consumption has already increased large levels of circulating MCP-1 in obese individuals, but a low glycemic index diet reduced them and had an impact via parathyroid hormones [68,69].

#### 3.2.6. C-Reactive Protein

C-reactive protein (CRP) is a major factor that has been associated with obesity. A growing body of evidence suggests that CRP may be more than merely an inflammatory measure. Adiposity development may be a key element in the generation of CRP and systemic inflammation that led to obesity. Increased CRP impacts blood counts for neutrophils, lymphocytes, monocytes, eosinophils, and basophils, as well as complement component levels in the classical pathway. It also noticeably alters the structure of the spleen, the largest lymphoid organ, where the number of white pulps (reservoirs of T- and B-lymphocytes) increased by about 2.5 times. Furthermore, CRP displays impressive regulation of the key proteins regulating energy expenditure [63,67].

#### 3.2.7. Plasminogen Activator Inhibitor 1

A physiological inhibitor of vitronectin and plasminogen activators (urokinase and tissue types) is the plasminogen activator inhibitor (PAI)-1. It is produced by adipose tissue, and obesity causes its plasma levels to rise; weight loss causes them to fall. Ectopic fat depots produce PAI-1 while being induced by inducers. Inflammation, oxidative stress, and circadian clock proteins are some of the recent inducers that have been identified. Through its indirect effects on insulin signaling, its influence on adipocyte differentiation, and its control over the recruitment of inflammatory cells within adipose tissue, PAI-1 may play multiple roles in the development of obesity. Obesity is caused due to an increase in PAI-1 secretion from adipose tissue, and this increase is correlated with the lipid composition and cell volume of fat cells. At least in part because of increased adipocyte gene expression, which in turn increases PAI-1 secretion from adipose tissue, plasma PAI-1 activity is raised in obesity [63,67,70].

### 3.3. The Interplay between Obesity, Oxidative Stress, Inflammation, and Cognitive Impairments

Oxidative stress and inflammation in obesity are closely associated with cognitive decline. For instance, high leptin concentration in the serum stimulates adipose tissue to release proinflammatory factors, such as IL-1, IL-6, and TNF, which may further promote the adipocytes to release more and more leptin [71]. IL-1 modulates hippocampal-dependent memory in the brain. The overexpression of IL-1 could induce microglial cells to exhibit inflammation by triggering morphological changes in the microglial cells via a process known as inflammatory microgliosis. Increased infiltration of the microglial facilitates neuroinflammation in the brain. In the dental gyrus, IL-1 plays a vital role in LTP, and excessive levels of IL-1 may block the regulatory action of IL-1 in learning and memory, causing memory decline. The expression of IL-1 in the hippocampus will inhibit the normal neurogenesis mechanism, leading to neuronal apoptosis, which may evince a depressive-like behavior [72]. Meanwhile, the excessive release of IL-6 will increase the formation of the vascular endothelial growth factor (VEGF). In such conditions, the VEGF enhances the release of nitric oxide synthase in the endothelial, which may suppress occludin, a tight junction protein present in the blood–brain barrier (BBB). A low concentration of occludin may cause the BBB to be more permeable [73], causing the upregulation of BACE1 enzymes in the brain. BACE1, in turn, may activate several underlying mediators that result in neuronal apoptosis and memory impairments [74]. Along with this, TNF-α is associated with hippocampal-dependent memory in the brain. TNF-α is also known for its neuromodulating activity in the brain, specifically by activating astrocytes and microglial activity. In this context, TNF-α impairs contextual fear memory by inhibiting the transmission of excitatory synaptic in the astrocytes. Simultaneously, TNF-α may inhibit LTP, a synaptic interaction between hippocampal neurons, which is responsible for learning and memory in the brain [75].

The underlying molecular pathway identified involved in TNF-α mediated spatial memory decline is via the cAMP response element binding protein (CREB) [75]. The CREB regulates the genes responsible for memory such as c-FOS, BDNF, and activity-dependent cytoskeleton-associated protein (Arc) in the hippocampus. The activation of c-FOS, BDNF, and Arc by the CREB is known to enhance neuronal survival and promote neurogenesis in the hippocampus, thereby improving spatial and fear-conditioning memory [76]. In obesity, the hippocampal level of CREB and the pro-inflammatory cytokine, such as IL-2, is suppressed [77]. The deterioration of IL-2 could induce cytoarchitectural changes in the hippocampus, such as impairing neurite branching and preventing the development of dendrites, eventually leading to memory decline [78]. The overexpression of IL-8 and monocyte chemotactic protein 1 (MCP-1) via the activation of NF-κB and its downstream mediators is another common implication of obesity-associated inflammatory changes in the hippocampus [79]. IL-8 is a pro-inflammatory factor that can cross the BBB, promote chemotaxis, and discharge lysosomal, and is mostly expressed in the microglial cells. In such conditions, microglial cells will secrete a toxic molecule known as glutamate, thereby inducing neuroinflammation in the brain. Resultantly, the synaptic transmission will be disrupted leading to neuronal dysfunction, and eventually cognitive decline [80]. Additionally, MCP-1 is known to promote microglial accumulations in the hippocampus [81], ergo promoting a condition known as “immune-vigilant”. The hippocampal region is more prone to neuroinflammation and the development of cognitive distortion [82].

**Figure 1:**
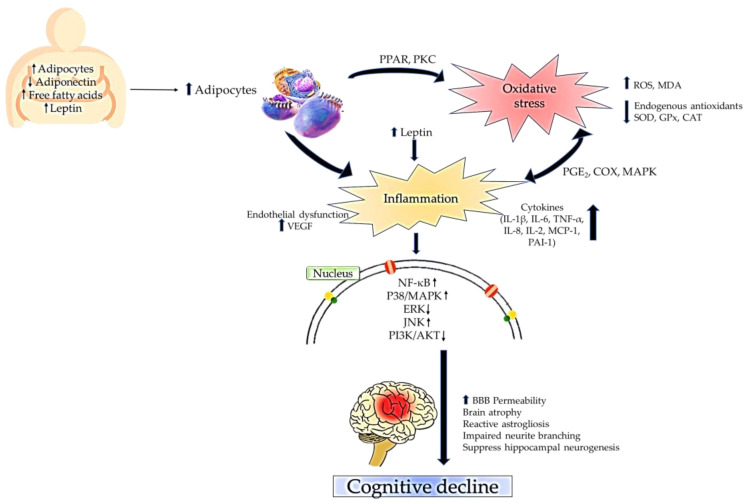
The interplay between obesity, oxidative stress, inflammation, and cognitive impairments.

The inflammatory nature of obesity could enhance the activity of lactate dehydrogenase (LDH) [83], CRP [84], and plasminogen activator inhibitor 1 (PAI-1) [85]. The overexpression of LDL in neurons enhances lactate oxidation, thereby stimulating the production of mitochondrial ROS, subsequently leading to neurodegeneration and memory impairment [86]. In accordance with this statement, a high level of CRP is an indicator of the increase in BBB permeability, the presence of a lesion in the white matter, an enlarged lacunar, and perivascular spaces in the brain. These morphological changes in the brain make the brain prone to neuroinflammation and neurodegeneration, indicating the marker for cognitive distortion [87]. Meanwhile, the elevation of PAI-1 impairs the maturation process of the BDNF in the neurons through the JNK/c-Jun signaling mechanism, thereby making the brain more susceptible to neurotoxicity. In such cases, one of the observable clinical symptoms will be cognitive decline [88]. In addition to this, the accumulation of pro-inflammatory lipid mediators, such as PGE_2_, is positively associated with cognitive impairments. The COX-induced inflammatory activity in the hippocampus and reactive glial cells can be detected through the increased level of PGE_2_ in the cerebrospinal fluid, which subsequently will manifest as cognitive decline [89]. Another common pathway involved in obesity-induced cognitive decline includes MAPK with downstream signaling involving ERK, JNK, and P38 MAPK. This pathway is often associated with cellular stress that disrupts insulin signaling in the brain. This will activate TNF-α mediated neuronal apoptosis in the hippocampus, resulting in cognitive impairments [90]. Figure 1 summarizes the interplay between obesity, oxidative stress, inflammation, and cognitive impairments.

## 4. The Association between Oxidative Stress and Endogenous Antioxidants in Obesity

Persistent inflammatory conditions seen in obesity can suppress endogenous antioxidants such as superoxide dismutase (SOD), catalase (CAT), and glutathione peroxidase (GPx) in the body. This is because excessive levels of adipokines secreted by the adipocytes will enhance the production of ROS, eventually leading to oxidative stress. Hypoxic conduction triggered by hyperplasic adipocytes further induces oxidative stress. This oxidative stress will depress the antioxidants, and one of the primary observable pathologies includes the increased concentration of malondialdehyde (MDA), a marker indicating cell damage, particularly in lipid peroxidation [14]. Additionally, the process of triglycerides and fatty acid synthesis known as lipogenesis is said to be markedly increased in obesity [68]. In such circumstances, lipogenesis via the pentose phosphate signaling mechanism will initiate the formation of NADPH oxidase [69]. NADPH oxidases expressed in macrophages will further lead to the formation of ROS. This happens during the catalytic activity of NADPH oxidase, which permits the electron transfer from NADPH, causing it to be reduced to O_2_^−^ by losing an oxygen molecule. Simultaneously, the superoxide anion will be transformed into an oxidizing agent known as H_2_O_2_. H_2_O_2_ will then be dissociated into H_2_O by either CAT or GPx. In case of antioxidant deficiency, the reaction between O_2_^−^ and H_2_O_2_, will result in the formation of ROS [66].

The formation of ROS in large quantities will eventually oxidize lipids, proteins, and mitochondrial DNA [63], causing the SOD level to decrease. In fact, a high level of calorie intake in obesity will cause the mitochondrial to be dysfunctional due to substrate load, eventually leading to the dissipation of the electron transfer or proton gradient, resulting in the formation of superoxide anions [67]. In addition, the presence of high triglyceride content could prevent the translocation of adenine nucleotides into the mitochondrial respiratory chain, triggering the formation of superoxide anion [64]. These changes will manifest as reduced endogenous antioxidants in obese subjects. A decreased level of GPx is one of the hallmarks of a defect in the mitochondrial oxidative phosphorylation system in adipose tissue [65]. In such circumstances, the oxidative capacity is diminished, promoting the formation of excessive ROS and leading to the suppression of GPx and CAT activity.

## 5. Conclusions

In conclusion, the greater amount of fat stored in individual adipocytes in obesity could induce the formation of ROS and inflammation. This will induce the generation of oxidative stress, which may cause morphological changes in the brain, suppress the endogenous antioxidant system, promote neuroinflammation, and eventually lead to neuronal apoptosis. This will impair the normal function of the brain and specific regions that are involved in learning and memory. Hence, obesity has a strong positive correlation with cognitive impairments. Therefore, this review may serve to provide a precise understanding of the mechanism linking obesity to the development of memory loss and might give an insight into therapeutic development focusing on oxidative stress and inflammatory pathways to manage an obesity-induced cognitive decline in the future.

## 6. Future Directions

A key future direction in the study of cognitive impairment in obesity is to investigate the specific mechanisms by which oxidative stress and inflammation contribute to these impairments. This should involve examining the roles of specific cellular and molecular pathways in order to develop a more detailed understanding of the underlying biology. Additionally, identifying potential therapeutic targets for reducing oxidative stress and inflammation in the brain could lead to more effective prevention and treatment strategies for obesity-related cognitive decline in a clinical approach. Longitudinal studies are also needed to examine the relationship between obesity, oxidative stress, inflammation, and cognitive decline in future clinical studies. This will be important for clarifying the temporal sequence of events and identifying potential early warning signs or risk factors for cognitive decline in individuals with obesity. Furthermore, lifestyle interventions, such as exercise and diet, may play a critical role in reducing oxidative stress and inflammation and improving cognitive function in individuals with obesity. Understanding the potential role of these interventions can help researchers develop more effective strategies for promoting healthy lifestyle behaviors and preventing or reducing the risk of cognitive decline in individuals with obesity. Overall, investigating the mechanisms of oxidative stress and inflammation in cognitive decline, identifying therapeutic targets, conducting longitudinal studies, and exploring the role of lifestyle interventions are important future directions for research in this area, specifically in human trials. A better understanding of the relationship between obesity, oxidative stress, inflammation, and cognitive decline may ultimately lead to the development of more effective prevention and treatment strategies for individuals with obesity.

## 7. Potential Implications

The potential implications of this narrative review are significant, both in terms of the prevention and treatment of cognitive decline in individuals with obesity. One important implication is the development of new therapeutic approaches that target oxidative stress and inflammation in the brain. By identifying specific cellular and molecular pathways involved in this process, researchers may be able to develop more targeted and effective treatments to prevent or slow cognitive decline in individuals with obesity. Additionally, the findings of this review underscore the importance of managing obesity as a potential way to prevent or reduce the risk of cognitive decline. This highlights the need for public health campaigns and policies aimed at promoting healthy weight management and reducing obesity rates. Understanding the mechanisms by which obesity contributes to cognitive decline may also help to identify individuals at higher risk and develop targeted prevention strategies. For example, identifying early warning signs of cognitive impairment may allow for earlier intervention and treatment. In conclusion, the potential implications of this review are significant and may lead to the development of more effective prevention and treatment strategies for cognitive decline in individuals with obesity based on animal model studies.

## 8. Strengths and Limitations

The strength of this study is the usage of a sensitive search strategy that has potentially identified most of the in vivo research articles that are relevant to this narrative review. However, there are still some limitations to our study, as our study is limited to in vivo study, and is also limited to pre-clinical data. Although in vivo trials are vital for preclinical research, this outcome could not entirely represent the pre-clinical reviews. In addition, our search was limited to research articles published in the English language. This may have missed relevant data published in languages other than English and could have introduced some sort of bias in the article selection. Another limitation is the timeframe the authors used in this search. Since the search strategy was limited to the past 10 years, we might have missed out on some important research outcomes that have been published beyond 10 years. Finally, some of the reporting assessments or the study included in this review may not be generalized to other forms of preclinical reviews.

## Data Availability

Not applicable.
